# Effects of cardiopulmonary bypass with low-priming volume on clinical outcomes in children undergoing congenital heart disease surgery

**DOI:** 10.1186/s13019-020-01151-w

**Published:** 2020-05-27

**Authors:** Lei Wang, Qiang Chen, Yong Qiang Qiu, Jian Xi Ye, Jian Zhi Du, Xiao Chai Lv, Yan Ting Hou, Liang Wan Chen

**Affiliations:** Department of Cardiac Surgery, Union Hospital of Fujian Medical University, Fuzhou, Fujian Province China

**Keywords:** Cardiopulmonary bypass, Low-priming volume, Children, Congenital heart disease surgery

## Abstract

**Background:**

Cardiopulmonary bypass (CPB) with high-priming volume can significantly activate the inflammatory response and increse the usage of packed red blood cells (PRBCs). As risks and complications related to transfusions are increasing, many cardiac centers are focusing on reducing the priming volume of CPB. In our center, efforts have also been made to reduce the priming volume, and the effects of CPB with low-priming volume on clinical outcomes in children undergoing congenital heart disease (CHD) surgery were investigated in this study to provide referential experiences for pediatric CPB.

**Methods:**

The clinical case data of 158 children undergoing CHD surgery with CPB were collected. The children were divided into the low-priming-volume group (group A, *n* = 79) and the traditional group (group B, *n* = 79) according to the priming volume. The amount of PRBCs transfused, the postoperative hematological test results and the clinical outcomes of the two groups were compared by the independent sample *t*-test or the chi-square test.

**Results:**

The amount of PRBCs transfused during CPB and during the whole operation were significantly lower in group A than in group B (*p* < 0.01), but the hemoglobin (Hb) concentration was higher in group A on the first day after surgery (*p* < 0.01) and before hospital discharge. However, the latter showed no statistical significant difference. The lowest postoperative platelet count was higher in group A than in group B (*p* < 0.05). There was no statistical difference in the postoperative inflammatory markers and the main clinical outcomes between the two groups.

**Conclusions:**

The usage of PRBCs in CPB with low-priming volume decreased significantly, but the postoperative Hb concentration and platelet count could still be maintained at a high level, improving the use efficiency of PRBCs. CPB with low-priming volume did not affect the postoperative recovery of patients, so it is worthy of continuous promotion and optimization.

## Introduction

Congenital heart disease (CHD) surgery in children accounts for a large part of cardiac surgery. The pathophysiological changes during cardiopulmonary bypass (CPB) in children are quite different from those in adults [1]. Children have a small body weight and blood volume, so the priming volume of CPB is a relatively large volume compared with adults. This means that the contact between the blood and the non-endothelial biomaterial surface increases, which activates the inflammatory response and coagulation system significantly substantially. Animals studies shows that when the priming volume been decreased by 50%, the levels of the inflammatory markers of TNF-α and IL-6 are lowered during CPB at 60 min [2]. On the other hand, the increased priming volume results in significant hemodilution and leads to increase the usage of PRBCs. However, the risks and complications related to transfusions are increasing [3–6]. Transfusions are even associated with increased mortality, becoming an independent risk factor of postoperative mortality [4]. Therefore, it is particularly important to reduce the priming volume of CPB, and at present more and more cardiac centers are focusing on it to alleviate the series of non-physiological reactions described above and reduce the use of blood products [7, 8]. But as a matter of fact there are no uniform guidelines and standards for pediatric CPB nowadays, different cardiac centers or even different perfusionists in the same center have different experiences [9]. Miniaturizing CPB tubes is the most important way to decrease the priming volume, but whether the CPB with low-priming volume is always better is uncertain. In our center, since 2016, we have reduced the priming volume of CPB by reducing the lengths and the inner diameters of the CPB tubes, and since 2019 a membrane oxygenator integrated with a microembolus filter has been used to further reduce the priming volume. We want to analyze the results of our work and guide future work further as well as provide referential experiences for pediatric CPB. So this study retrospectively analyzed the effects of CPB with low-priming volume on clinical outcomes in children undergoing CHD surgery.

## Methods

### Grouping

The clinical case data of 158 children undergoing CHD surgery with CPB in our hospital from May 2017 to August 2019 were collected. According to the priming volume of CPB, the patients were divided into the low-priming-volume group (group A, *n* = 79) and the traditional group (group A, *n* = 79).

### Diagnostic criteria, inclusion criteria and exclusion criteria

Diagnostic criteria: All patients were clearly diagnosed according to clinical manifestations, echocardiography and chest radiographs. Inclusion criteria: Patients with a body weight ≤ 15 kg and the priming volume of CPB was 110 to 350 ml; patients who had undergone selective uncomplicated CHD surgery with CPB and the risk adjustment in congenital heart surgery (RACHS-1) category was 1 to 2; the hemodynamics before surgery was stable. Exclusion criteria: Transfusion of PRBCs was required because of severe anemia preoperatively; secondary thoracotomy or endotracheal intubation was performed postoperatively; early clinical death occurred within 48 h after surgery.

### Main clinical outcomes

The main clinical outcomes included the usage rate and amount of PRBCs perioperatively, the changes of hemoglobin (Hb) concentration, platelet count and inflammatory markers including the maximum values of white blood cell (WBC) number, eutrophils percentage, C-reactive protein (CRP), blood glucose, body temperature, the incidence of pneumonia, the urine volume and thoracic drainage fluid 48 h after surgery, the postoperative mechanical ventilation time, the length of ICU stay and the postoperative stay in the general ward.

### The priming of CPB

**Fig. 1:**
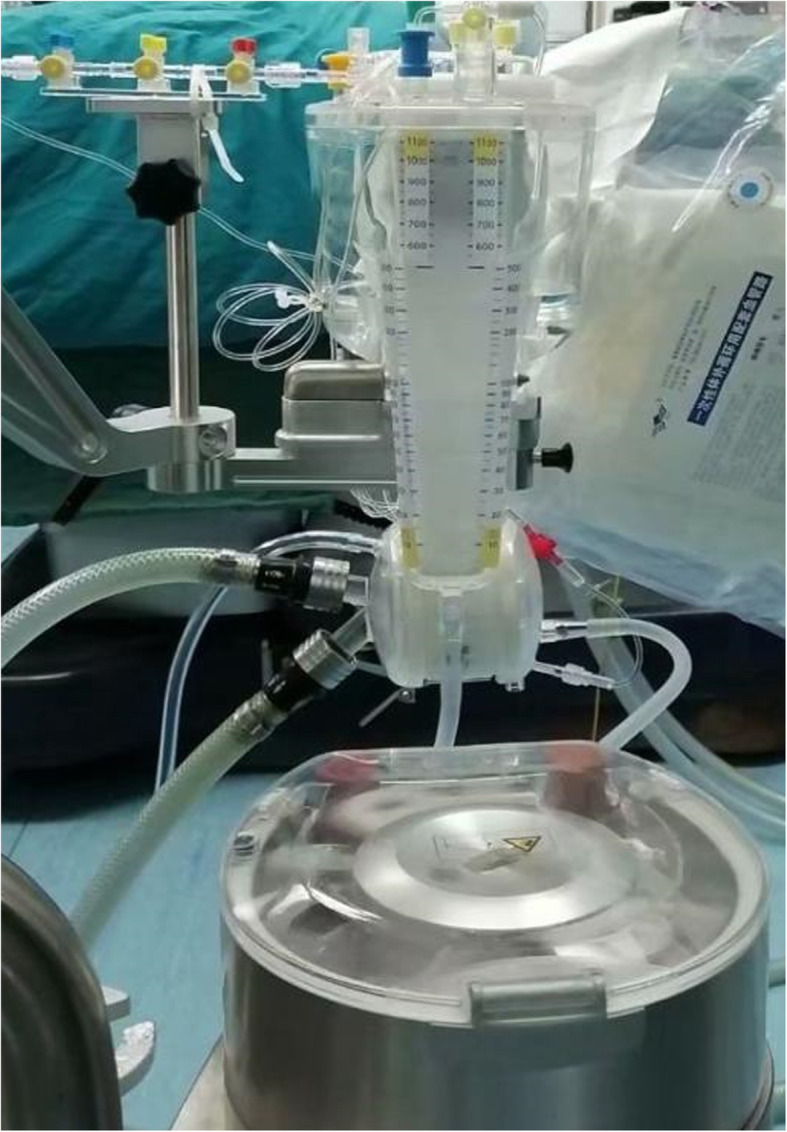
The CPB with low-priming volume. The lowest priming volume was 110 ml in our study by using a microembolus-filter integrated oxygenator and shorter and thinner tubes. The oxygenator was located close to the pump and the operating bed

A Stockert type III or V CPB machine was used. The priming volumes of CPB, the lengths and the inner diameters of the CPB tubes of the two groups are shown in Table 1. The lowest priming volume of CPB was 110 ml, as shown in Fig. 1. In group A, the priming volume was 110 ~ 180 ml. The membrane oxygenator used included two types, the Terumo RX05 and the Terumo FX05. The latter oxygenator was integrated with a microembolus filter not requiring an additional one, so it reduced the priming volume by about 65 ml. The inner diameters of the CPB tubes used in children with body weights≤10 kg were gradient, that is, the diameters of the venous tube and the tube in the main pump were 1/4 in., and the diameters of the rest tubes were 3/16 in.. On the other hand, the oxygenator was located close to the main pump and the operating bed with the shortened tubes. Under this circumstances, the oxygenator was almost at the same height as the operating bed, reducing the venous drainage assisted by gravity. So a vacuum-assist venous drainage (VAVD) controller was needed to facilitate venous drainage with a negative pressure from − 20 mmHg to − 40 mmHg. In group B, like group A, two types of oxygenators was used, but the lengths of the CPB tubes were longer, and the inner diameters of the CPB tubes were all 1/4 in. regardless of the weights of children. So the priming volume in group B with a volume of 210 ~ 350 ml was more than that in group A, and the oxygenator was located at a certain height and distance from the operating bed, with venous drainage assisted by gravity effectively. In the both groups, the priming liquid used was compound sodium chloride, and an appropriate amount of PRBCs was used in the priming of CPB according to the patient’s body weight and preoperative Hb concentration. Some of the patients whose body weights ≥8 kg and whose preoperative Hb concentrations ≥11 g/dL were treated with bloodless priming, using Voluven or 20% albumin to replace the most initial priming liquid. A moderate amount of plasma was transfused during CPB in group B.

**Table 1:** The priming volume of CPB, the lengths and the inner diameters of the CPB tubes

Group	Body weight	Membrane oxygenator	Priming volume (ml)	Length of CPB tubes (cm)	Diameter of arterial tube (inches)	Diameter of venous tube (inches)	Diameter of main pump tube (inches)	Diameter of the rest CPB tubes (inches)
group A	≤10 kg	FX05	110–120	255	3/16	1/4	1/4	3/16
		RX05	150–160	320	3/16	1/4	1/4	3/16
	10–15 kg	FX05	130–140	255	1/4	1/4	1/4	1/4
		RX05	170–180	320	1/4	1/4	1/4	1/4
group B	≤15 kg	FX05	210–300	395	1/4	1/4	1/4	1/4
		RX05	250–350	460	1/4	1/4	1/4	1/4

*CPB* cardiopulmonary bypass

### Management during CPB

All patients underwent general anesthesia with endotracheal intubation. A shallow low temperature and high flow perfusion was adopted during CPB. The cardioplegic solution was histidine-tryptophan-ketoglutarate solution with a dose of 40 ~ 60 ml/kg. The conventional ultrafiltration was performed to remove excess fluid and to increase the Hb concentrations. The lowest acceptable Hb concentration during CPB was 7 g/dL, and before CPB weaning the Hb concentration should be greater than 9 g/dL. In group A, not all PRBCs prepared in advance were transfused during CPB. When the Hb concentration reached the standard of CPB weaning, the remaining PRBCs were transfused through a peripheral vein by the anesthesiologist after CPB weaning. Unlike this, in group B, almost all PRBCs prepared were transfused during CPB. The mean arterial pressure, central venous pressure, mixed venous oxygen saturation (SvO_2_), negative pressure in the reservoir, blood gas analysis, activity coagulation time and nasopharyngeal temperature were continuously monitored during CPB, and all parameters were adjusted within the normal range.

### Statistical methods

Statistical analysis was performed with SPSS version 25. Descriptive analysis was carried out for each variable. The measurement data were expressed as ($\overline{\mathrm{x}}$±S), and an independent sample *t*-test was used to compare the measurement data between the two groups. The enumeration data were expressed as the frequency or rate, and the chi-square test was used to compare the enumeration data between the two groups. Values of *p* < 0.05 were considered to be statistically significant.

## Results

### Comparison of the preoperative clinical data between the two groups

As shown in Table 2, there were no significant differences between the two groups in terms of the preoperative data.

**Table 2:** Comparison of the preoperative clinical data between the two groups

Preoperative data	Low-priming-volume group (group A, *n* = 79)	Traditional group (group B, *n* = 79)	*p* value
male/case	48	48	
age/month (range)	1 ~ 48 (13.66 ± 12.59)	1.5 ~ 48 (14.51 ± 13.31)	0.681
body weight/kg (range)	4 ~ 15 (8.62 ± 3.31)	4 ~ 15 (8.91 ± 3.35)	0.859
main diagnostic/case			
ASD(+PDA)	7	3	
ASD + PS(+PDA)	9	7	
VSD(+PDA)	29	28	
VSD + PS	5	0	
VSD + ASD(+PDA)	27	29	
PS	0	5	
PAPVC+ASD	1	4	
PECD	0	1	
TOF	1	2	
EF/%	69.46 ± 6.19	69.88 ± 6.97	0.689
WBCs/(10*9/L)	9.13 ± 2.67	8.64 ± 2.48	0.240
neutrophil percentage/%	29.49 ± 12.28	31.91 ± 12.55	0.230
Hb/(g/dL)	11.88 ± 1.67	12.18 ± 1.81	0.280
platelets/(10*9/L)	338.62 ± 89.67	332.72 ± 110.86	0.718
CRP/(mg/L)	0.93 ± 1.95	0.62 ± 0.95	0.198
blood glucose/(mmol/L)	4.68 ± 0.81	4.68 ± 0.88	0.950
total bilirubin/(μmol/L)	7.17 ± 5.27	9.01 ± 9.27	0.134
creatinine/(μmol/L)	24.31 ± 8.95	24.41 ± 6.08	0.937

*ASD* atrial septal defect, *PDA* patent ductus arteriosus, *PS* pulmonary stenosis, *VSD* ventricular septal defect, *PAPVC* partial anomalous pulmonary venous connection, *PECD* partial endocardial cushion defect, *TOF* tetralogy of Fallot, *EF* ejection fraction, *WBC* white blood cell, *Hb* hemoglobin, *CRP* C-reactive protein

### Comparison of the intraoperative clinical data between the two groups

As shown in Table 3, the priming volume of CPB and the PRBCs used in the priming of CPB were significantly lower in group A than in group B (*p* = 0.000). The total amount of PRBCs transfused during CPB and during the whole operation was significantly lower in group A than in group B (*p* < 0.01). The Hb concentration during CPB and before CPB weaning was lower in group A than in group B (*p* < 0.01).

**Table 3:** Comparison of the intraoperative clinical data between the two groups

Intraoperative data	Low-priming volume group (group A, *n* = 79)	Traditional group (group B, *n* = 79)	*p* value
CPB time/min	72.41 ± 23.10	73.22 ± 28.25	0.844
aortic cross-clamping time/min	34.54 ± 22.09	35.01 ± 21.89	0.894
priming volume of CPB/ml	170.63 ± 14.70	274.55 ± 48.96	0.000**
PRBCs used in the priming of CPB/ml	34.23 ± 23.93	108.04 ± 57.17	0.000**
CPB with booldless priming/case(%)	26 (32.9%)	12 (15.2%)	0.009**
CPB with no transfusion/case(%)	11 (13.9%)	7 (8.9%)	0.483
total amount of PRBCs transfused during CPB/ml	189.11 ± 101.47	244.30 ± 96.41	0.001**
PRBCs transfused by the anaesthetists/ml	11.54 ± 37.73	1.90 ± 16.87	0.042*
PRBCs transfused during the whole operation/ml	200.51 ± 101.74	246.20 ± 96.00	0.004**
Hb during CPB/(g/dL)	8.95 ± 1.15	9.93 ± 1.92	0.000**
Hb before CPB weaning/(g/dL)	11.51 ± 1.33	12.25 ± 1.78	0.003**

*CPB* cardiopulmonary bypass, *PRBCs* packed red blood cells, *Hb* hemoglobin

*was *p* < 0.05, **was *p* < 0.01

### Comparison of the postoperative clinical data between the two groups

As shown in Table 4, the Hb concentration was higher in group A than in group B on the first day after surgery (*p* = 0.000) and before hospital discharge (the latter had no statistically significance). The lowest postoperative platelet count was higher in group A than in group B (*p* < 0.05), but none of the two groups met the standard of platelet transfusion. There were no statistically significant differences between the two groups in terms of the inflammatory markers, the incidence of pneumonia, the value of total bilirubin or creatinine, the volume of urine or thoracic drainage fluid 48 h after surgery. And there were also no statistically significant differences between the two groups in terms of the mechanical ventilation time, the lengths of ICU stay or the postoperative stay in the general ward. There were no positive blood culture, serious liver or kidney dysfunction, neurological complications or deaths in either group postoperatively.

**Table 4:** Comparison of the postoperative clinical data between the two groups

Postoperative data	Low-priming-volume group (group A, *n* = 79)	Traditional group (group B, *n* = 79)	*p* value
Hb on the first day after surgery/(g/dL)	13.12 ± 1.75	12.07 ± 1.67	0.000**
Hb before hospital discharge/(g/dL)	11.53 ± 1.45	11.22 ± 1.61	0.199
lowest value of platelet count/(10*9/L)	208.85 ± 70.07	186.38 ± 58.26	0.030*
highest value of WBCs/(10*9/L)	13.55 ± 3.82	12.72 ± 4.34	0.206
highest value of neutrophil percentage/%	76.42 ± 8.48	76.57 ± 10.51	0.923
highest value of CRP/(mg/L)	49.86 ± 41.09	40.12 ± 32.47	0.143
highest value of lactic acid/(mmol/L)	2.30 ± 1.48	2.32 ± 1.40	0.916
highest value of blood glucose/(mmol/L)	12.40 ± 6.07	12.07 ± 5.09	0.722
highest value of temperature/°C	38.20 ± 2.28	38.51 ± 0.51	0.239
highest value of total bilirubin/(μmol/L)	18.00 ± 14.28	19.14 ± 14.37	0.630
highest value of creatinine/(μmol/L)	32.67 ± 9.64	34.58 ± 14.96	0.361
incidence of pneumonia			
unilateral pneumonia/case (rate)	24 (30.4%)	21 (26.6%)	0.597
bilateral pneumonia/case (rate)	28 (35.4%)	26 (32.9%)	0.737
thoracic drainage fluid 48 h after surgery/ml	98.23 ± 54.83	93.54 ± 48.44	0.570
urine volume 48 h after surgery/ml	825.50 ± 265.90	774.73 ± 297.42	0.384
mechanical ventilation time/h	26.26 ± 35.28	25.16 ± 37.81	0.850
length of ICU stay/d	2.50 ± 2.48	2.55 ± 2.29	0.910
length of postoperative stay in the general ward/d	8.26 ± 4.56	8.22 ± 3.17	0.939

*was *p* < 0.05, **was *p* < 0.01

*Hb* hemoglobin, *WBC* white blood cell, *CRP* C-reactive protein, *PRBCs* packed red blood cells

## Discussion

In our study, the cases were all children undergoing simple CHD surgery, and children undergoing complicated CHD surgery or in a critical condition were excluded as their severe illness may be accompanied by other disturbances and may result in a lower transfusion threshold.

CPB equipment determines the priming volume. Improving CPB equipment is the most fundamental measure to reduce the priming volume, and miniaturizing CPB tubes is the most important one. In this study, the lowest priming volume of CPB was 110 ml by reducing the lengths and the inner diameters of the CPB tubes and by using the oxygenator integrated with a microembolus filter. These measures significantly reduced the priming volume, but the following situations also need to be noted. Firstly, the distance and height of the oxygenator were located close to the operation bed weakening the venous drainage via gravity and leading to edema of brain and abdominal viscera probablely. So the VAVD controller played an important role as it promotes the venous drainage. When a VAVD controller is used, a thinner venous tube can be used, which is beneficial for reducing the priming volume of CPB [10]. During CPB the gas-blood mixed liquid is suctioned into the reservoir of the oxygenator, leading to a reduction of negative pressure in the venous tube and in the inlet of the reservoir [11]. As a result, the negative pressure in the reservoir changes. Therefore, we routinely monitored the negative pressure in the reservoir, and the monitored value was equivalent to the set value. A high level of negative pressure may cause hemolysis. Usually, the highest negative pressure is − 60 mmHg [12]. For the patients under 10 kg, the VAVD negative pressure of (− 20 ~ − 60) mmHg do not change the degree of hemolysis [12]. Another complication associated with the VAVD controller is that the bubble from the oxygenator precipitate into the blood, leading to gas embolism. However, in our study, no visible hemoglobinuria or obvious increases in bilirubin were observed in either group, and no obvious organ disfunctions were observed postoperatively. Therefore, the VAVD remains an important auxiliary means for reducing the priming volume of CPB in pediatric cardiac surgery. Secondly, in addition to venous drainage, arterial perfusion should also be noted. In our study, the inner diameter of the artery tube was 3/16 in. in children with a body weight less than 10 kg. But a study shows that the priming volume of CPB might be reduced slightly while the arterial pressure is increased, leading to the possibility of hemolysis [13]. Besides, the increased circulation resistance might result in a loss of hemodynamic energy delivered to patients, leading to a drop in the blood pressure of patients [13]. But in our study, patients had normal blood pressure and mixed SvO2 and had no significant increase in lactate levels, indicating that tissue perfusion is sufficient. Thirdly, in the low-priming-volume group, the oxygenator was located close to the operation bed and the patient’s head, which limited the operating space of the assistant but did not affect the surgeon. Not all cardiac surgeons were willing to accept this limitation. Fourthly, when the CPB tubes was located close to the operation bed, the aseptic principle was easily violated. However, our study results showed the patients in the low-priming-volume group demonstrated no increase in the maximum postoperative temperature, and there was no positive blood culture in either group. Therefore, as long as the principles of the aseptic manipulation are strictly observed, CPB with low-priming volume does not increase the incidence of postoperative infection.

The contact between blood and biomaterial surface of CPB circuit results in platelet adhesion, WBC and coagulation system activation. An activated systemic inflammatory response syndrome leads to systemic organ dysfunction in pediatric patients and prolongs the length of ICU stay for 3 days [14]. Animal studies shows that decrease CPB surface area and the priming volume can decrease the levels of the inflammatory markers during CPB at 60 min, but there is no difference during CPB at 120 min. Therefore, the release of inflammatory markers during CPB is associated not only with the size of the contact area between the blood and biomaterial surface but also with the CPB time [2]. In our study, the inflammatory markers of the highest values of WBC number, the neutrophil percentage and CRP showed no significant difference between the two groups. The reason might be that although there was a significant difference in priming volume between the two groups, such a difference could not cause a significant difference in inflammatory response. The minimum priming volume found in an advanced heart center was only 73 ml [7], so there is still much room for improvement in our equipment to reduce the priming volume. In addition, a Terumo membrane oxygenator with a hydrophilic coating was adopted for all patients in our study, which could have improved the blood compatibility of biomaterials and reduced the activation of the inflammatory response [15]. Besides, in our study, albumin was primed in the reservoirs and was coated on the inner surface of reservoirs and tubes before CPB in order to reduce the adhesion of platelets and the activation of non-physiological reactions. All these factors might have reduced the difference in the value of inflammatory markers between the two groups.

Reducing the lengths and the inner diameters of the CPB tubes decreases the priming volume and blood transfusions [3, 8], and reduces the rate of utilization of extracorporeal membrane oxygenation (ECMO) in newborns postoperatively [8], and does not increase postoperative complications or mortality [16]. In the low-priming-volume group of our study, although the preoperative Hb concentration was lower, the amount of PRBCs usaged during CPB was lower and the postoperative Hb concentration was higher. This means that reducing the priming volume of CPB is the key to alleviating hemodilution, reducing PRBCs transfusions and maintaining a similar Hb concentration before hospital discharge in children. Studies shows that pediatric transfusions are associated with a prolonged postoperative mechanical ventilation time and lengths of ICU stay, a higher lactic acid level and an increased bleeding volume 48 h after surgery [3, 5, 17], and even become an independent risk factor for postoperative mortality [4]. Transfusions after CPB are less complicated than transfusions during CPB [17]. The reason is that transfusions during CPB make PRBCs easily form into microaggregates and the brittleness of RBCs membrane increases, which lead to microcirculation embolism easier; Blood transfusions cause an increase in the erythrocyte morphological index and a decrease in the deformation index during CPB [18]; The high shear force during CPB can easily damage RBCs and lead to hemolysis, and these conditions are also associated with transfusion-related lung injury [19]. Therefore, Boettcher Wolfgang believes that routine application of bloodless priming in neonatal open-heart surgery is safe and that delaying transfusion until the end of CPB is beneficial for overall restrictive transfusions [5]. And so did our study, some patients with a body weight of more than 8 kg and a preoperative Hb concentration of more than 11 g/dL were treated with bloodless priming. The amount of PRBCs used in the priming of CPB partially depended on the experience of perfusionists, and most importantly depended on the expected degree of hemodilution according to the priming volume of CPB, body weights and Hb concentrations preoperatively. The rate of bloodless priming was statistically significant lower in group B than in group A, dealing with significantly higher PRBC transfusion rates. The body weights and Hb concentrations were similar in the two groups, so the larger priming volume of CPB may be an important factor for more RBC tranfusion rates. During CPB, not all PRBCs were transfused while the Hb concentration reached the standard for CPB weaning. The remaining PRBCs were transfused from a peripheral vein by the anesthesiologists. This methods lead to a significant lower PRBCs transfusions and a higher Hb concentration postoperatively. And this result might be related to the decrease of transfusion-related complications and RBC destruction, suggesting that CPB with low-priming volume can reduce the usage of PRBCs and improve the use efficiency of PRBCs. There were no difference of the postoperative results between the two groups, possibly as these patients were not in critical illness and may not be susceptible to transfusions. There is another result need to be pay attention to. That is, the rate of bloodless priming of CPB was significant higher in the low-priming-volume group, but the number of patients without transfusions during the whole operation was still very rare. In our study, patients escaping from transfusions were those who had a heavier body weight and higher Hb concentrations preoperatively. Although at present, the effects of hemodilution on children are not fully understood, and the minimum safe hematocrit and the general accepted transfusion threshold during CPB in children have not been standardized, for patients not in critical illness and have a heavier body weight, the usage of PRBCs maybe too much in our study. Maybe some patients could have escaped from transfusions, but we perfusionist prepared too much PRBCs in advance preoperatively. So we need to decrease the usage of PRBCs in the low risk and simple cases in the future. Blood conservation is a team project, so in order to make significant progress in maintaining a blood reserve, perfusionists, surgeons, anesthesiologists, and especially perfusionists, need to be more proactive in reducing usage of blood products.

In this study, the lowest postoperative platelet count was significantly higher in the low-priming volume group than in the traditional group. The decrease in the platelet count of children after CHD surgery with CPB was related to hemodilution, the contact between the blood and biomaterial surface leading to platelet activation and consumption, the CPB time, the aortic cross-clamping time and the intraoperative temperature, etc [20]. Platelet dysfunction after cardiac surgery is an important factor for bleeding after surgery [21]. In our study, there was no significant difference in CPB time, aortic cross-clamping time or temperature during the aortic cross-clamping process between the two groups. Therefore, CPB with low-priming volume might be a protective factor for maintaining platelet count. However, in this study, there was no significant difference in postoperative thoracic drainage fluid 48 h after surgery between the two groups. The reason might be that although the platelet count was decreased, it was still in the normal range, thus reducing the influence on coagulation function, and a few patients with postoperative transfusions of plasma might have affected the analysis of the results.

This study had three limitations. Firstly, this is a retrospective study, so it may have sample selection bias. Secondly, patients with complicated CHD surgery or in a critical condition were excluded in our study, but the value of miniaturization of the CPB circuit maybe more meaningful in the more complex and smaller children, so this study is limited by the excluded patients, which requires us to study further. Thirdly, in our study, the number of patients without transfusions during the whole operation was still very rare. This is an unfortunate result which prevents us from further study due to the rare case of transfusion-free patients. So we need to decrease the usage of PRBCs in the low risk and simple cases in the future.

## Conclusions

The usage of the PRBCs during CPB with low-priming volume decreased significantly, but the postoperative Hb concentration and platelet count could still be maintained at a high level, improving the use efficiency of the PRBCs. CPB with low-priming volume did not increase the risk of hemolysis or the incidence of postoperative infection and did not affect the postoperative recovery of patients, so it is worthy of continuous promotion and optimization.

## Data Availability

The datasets during and/or analysed during the current study available from the corresponding author on reasonable request.

## Abbreviations

CPB: Cardiopulmonary bypass
CHD: Congenital heart disease
PRBCs: Packed red blood cells
Hb: Hemoglobin
EF: Ejection fraction
RACHS-1: Risk adjustment in congenital heart surgery
WBC: White blood cell
CRP: C-Reactive protein
ASD: Atrial septal defect
PDA: Patent ductus arteriosus
PS: Pulmonary stenosis
VSD: Ventricular septal defect
PAPVC: Partial anomalous pulmonary venous connection
PECD: Partial endocardial cushion defect
TOF: Tetralogy of Fallot
VAVD: Vacuum-assist venous drainage
SvO2: Venous oxygen saturation
ECMO: Extracorporeal membrane oxygenation
